# Flame-Retardant Performance Evaluation of Functional Coatings Filled with Mg(OH)_2_ and Al(OH)_3_

**DOI:** 10.3390/polym14030372

**Published:** 2022-01-18

**Authors:** Elpida Piperopoulos, Giuseppe Scionti, Mario Atria, Luigi Calabrese, Edoardo Proverbio

**Affiliations:** 1 Dipartimento di Ingegneria, Università di Messina, Contra di Dio-Sant’Agata, 98166 Messina, Italy; pepi_scionti@hotmail.com (G.S.); eproverbio@unime.it (E.P.); 2 Colorificio Atria, Contrada Camarro Formeca, 91028 Partanna, Italy; amario@atria.it

**Keywords:** flame-retardant coating, magnesium hydroxide, aluminum hydroxide

## Abstract

In the shipbuilding sector (cruises, ferries, etc.), the design and control constraints applied to improve the fire safety conditions of naval vessels are acquiring important relevance. Research activities have aimed at enhancing the fire resistance of structures and surface coatings to make ships’ working environments safer, trying to combine performance, durability and low costs. In this context, the aim of this paper is to develop and optimize flame-retardant coatings for naval applications. In particular, in an acrylic carrier, Mg(OH)_2_ and Al(OH)_3_ fillers were added to exalt the fire resistance capabilities of the coatings. Furthermore, the effect of the particle size of the hydroxides on the coatings’ fire resistance was investigated. The coatings were studied by structural (XRD), thermo-physical (TG) and morphological (SEM) characterization to evaluate their thermal stability and the damage level due to fire exposition. Specifically, fire reaction tests were applied at different fire exposure times (15 s, 30 s) to estimate the fire resistance of the proposed coatings compared to the commercial reference. The results show that the coatings based on aluminum and magnesium hydroxides exhibit favorable fire resistance. Particularly, effective performances were observed for short times of exposure to direct flames. Furthermore, the temperature monitoring of the steel alloy support during the test allowed us to evaluate the degree of insulation of the coating, highlighting a better result for the specimen filled with Mg(OH)_2_, making this product promising for its optimization in this context.

## 1. Introduction

In the shipbuilding field, the most common method to protect structures from atmospheric agents is to use protective coatings. In this context, particular attention must be paid to the fire resistance and safety of the coating in order to minimize structural, economic and human risks. Therefore, it is important to develop effective methods for fire protection to avoid possible accidents and to reduce the social and economic damages due to fire [1].

Fire triggering and propagation is a complex phenomenon that involves different stages: (i) initiation (e.g., heating due to activation of ignition); (ii) growth (e.g., spread or propagation of the flames extending the combustion areas); (iii) a steady state, where the fire has completely evolved; and finally, (iv) decay, where self-extinguishing occurs [2].

A passive approach to improving fire resistance is to apply barrier fillers that have effective components to increase both thermal insulation and fire stability. In this context, different types of inert fillers have been proposed with suitable results, such as silica [3], graphene oxide [4], carbon nanotubes [5] and glass [6].

To increase the thermal and fire resistance of the coatings by means of passive protection, promising and interesting results have been achieved by the use of hollow glass microspheres [7,8]. This approach has the advantage of preserving the fire resistance provided by the glass particles, as well as ensuring a significant improvement in thermal insulation.

One of the most applied active prevention and protection methods is the use of flame-retardant (FR) coatings. These coatings must be able to hinder the growth stage in order to preserve the structural stability and the functionality of the sites affected by the fire accident [9].

Since the polymer matrix, which acts as binder, belongs to flammable materials, the effectiveness of this property is crucial to ensure its installation and stability during its service life in multiple application contexts [10]. Usually, FR coatings are obtained by incorporating additives that contain halogens (e.g., chlorinated or brominated compounds) or phosphorous compounds, which usually help to inhibit flame spread by radical quenching or the formation on the surface of a glassy protective layer [11].

In such a context, hydrated inorganic compounds, e.g., aluminum hydroxide, (Al(OH)_3_) and magnesium hydroxide (Mg(OH)_2_), are suitable and halogen-free effective alternatives as flame-retardant additives.

Aluminum hydroxide is amphoteric: it acts as a base, forming a salt in acidic media, while in basic media, it acts as a Lewis acid. A peculiar feature useful in an FR additive is that Al(OH)_3_, at about 180 °C, decomposes with an endothermic dehydration reaction, releasing water vapor [12]. Analogously, Mg(OH)_2_ experiences a similar decomposition at 332 °C, absorbing large amounts of heat and releasing water vapor [13]. Furthermore, coupled to prevent polymer matrix combustion, these metal hydroxides act as effective smoke suppressants [14]. In particular, the involved decomposition reactions are the following:
$2Al{\left(OH\right)}_{3}\to Al_{2}O_{3}+3H_{2}O$1050 J/g [15](1)$Mg{\left(OH\right)}_{2}\to MgO+H_{2}O$1389 J/g [16](2)

A high heat of reaction characterizes the two reactions. Consequently, at the chosen temperature, the activation of the aforementioned endothermic reactions allows heat to be dissipated during exposure to fire, leading to a cooling effect on the polymer. At the same time, the water vapor, as a decomposition product, can dilute the polymerization of the flammable gas, hinder the combustion growth and absorb the smoke particles, leading to a smoke suppression effect. Nevertheless, high filler content is required, usually >60wt% (37 vol%) in polymer matrices, resulting in a significant loss of composite mechanical performance [13].

Liang et al., in [17,18], investigated the flame-retardant properties of a polypropylene-based composite coating with either aluminum hydroxide, magnesium hydroxide or zinc borate. The FR content was added in the range of 10 to 70 phr. The results highlight that the flame-retardant and smoke suppression effects of Al(OH)_3_ and Mg(OH)_2_ on the polymer matrix are significant. Furthermore, they observed that the burning speed decreases with increasing FR content, with the optimum at 70 phr. Analogous considerations were observed by Chen et al. [19] by using Mg(OH)_2_ as an FR, varying the content in the range of 10 to 60wt% in the composites.

However, high contents of flame retardants have harmful effects on the mechanical properties of the coating. For fire-resistant polymeric coatings, both flame-retardant properties and mechanical properties are design constrains that must be preserved [20].

Consequently, the search for formulations with lower flame-retardant contents can represent an effective strategy for acquiring a performance compromise between mechanical stability and fire resistance.

To this end, the development of a system that integrates active and passive fire protection can represent an effective strategy for obtaining a well-tailored coating. The aim of this paper is to compare the flame resistance properties of acrylic composite coatings. Low-content Mg(OH)_2_ and Al(OH)_3_ fillers were added as FRs in order to improve the fire resistance performances of the coatings. Commercial and laboratory-synthetized hydroxides were compared to better assess the influence of the filler morphology on the coatings’ performance. All samples were investigated by structural (XRD), thermo-physical (TG) and morphological (SEM) analysis to evaluate their thermal stability. Furthermore, fire reaction tests were carried out at different fire exposure times (15 s, 30 s) to evaluate the fire resistance capacity of the proposed formulations.

## 2. Materials and Methods

### 2.1. Materials

Investigated coatings were functionally filled to study the fire behavior and to evaluate a possible implementation of fire resistance capacity. Two kinds of fillers were added, aluminum hydroxide (Al(OH)_3_) and magnesium hydroxide (Mg(OH)_2_). Commercial and laboratory-synthesized materials were employed to identify two different morphologies and understand the influence of the surface structure on the coating performance. The commercial Al(OH)_3_ (C-AH) was purchased from Sigma Aldrich (reagent grade, St. Louis, MO, USA). The synthesized material was obtained by the precipitation method and is named S-AH. The following reagents were used as precipitating agents: aluminum nitrate hexahydrate (Al(NO_3_)_3_ ∙ 9H_2_O, 99wt%, Carlo Erba Reagents, Milan, Italy) and ammonium hydroxide (NH_4_OH, 30wt%, Honeywell Fluka, Charlotte, NC, USA). The synthesis was carried out under stirring, gradually adding, by means of a peristaltic pump (2.5 mL/min), 50 mL of a 0.9 mol/L NH_4_OH solution (pH = 11.8) to 100 mL of Al(NO_3_)_3_, 0.13 mol/L (final solution pH = 8.49), in order to obtain 3 g of precipitate. The obtained solution was left to stand at room temperature for 24 h and then filtered under a vacuum, using a filter with a thickness of 0.22 µm. The precipitate was then washed with deionized water and dried in an oven at 80 °C for one night. Pure Al(OH)_3_ was obtained.

**Figure 1:**
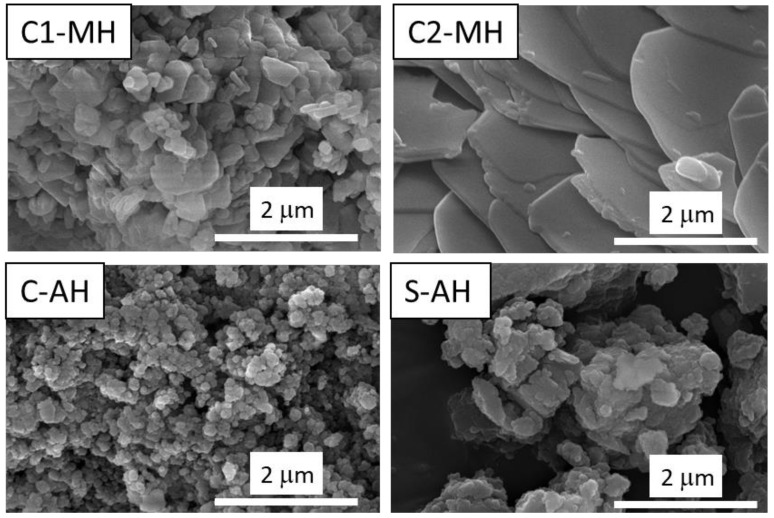
SEM images of the employed fillers, C1-MH, C2-MH, C-AH and S-AH.

Two commercial Mg(OH)_2_ fillers were purchased from Acros Organics B.V.B.A. (95wt%, Waltham, MA, USA) and Kisuma Chemicals BV (>98wt%, Veendam, The Netherlands), named C1-MH and C2-MH, respectively. The scanning electron microscopy (SEM) images of the above-mentioned materials are reported in Figure 1.

The samples show a different morphology. Two of the investigated fillers (C1-MH and C-AH) show a smaller particle size with distinct and homogeneous particles; in particular, the C-AH sample exhibits particles of 150 nm, on average. The S-AH sample, on the contrary, shows a larger particle size, characterized by a more inhomogeneous distribution. In addition, a packed morphology is noted, due to a noticeable coalescence between the material’s particles. A different aspect is highlighted by the C2-MH sample, in which plate-like particles, typical of brucite phase [21], with dimensions of about 4 µm, are observed.

### 2.2. Coating Preparation

The coatings, the objects of this study, were realized by the Colorificio Atria S.r.l. (Partanna, Italy) paint factory, adding the previously described fillers to a formulation based on an acrylic carrier. More additives are present in the basic formulation, such as TiO_2_, CaCO_3_ and hollow microspheres. Titania nanoparticles have a significant effect on decreasing the rate of flame spread [22]. CaCO_3_ is used to enhance the coating resistance to wear and scratching [23]. Hollow microspheres generally decrease bulk density and increase temperature insulation without compromising mechanical performance [24]. Moreover, the three fillers are usually used to help reflect solar energy back into the atmosphere, rather than heating up the structure. All formulations were deposited by using a BYK manual film applicator (coating thickness 770 μm) on a stainless steel support (dimension 75 mm × 140 mm × 0.8 mm). All realized samples are coded with two suffixes. The first suffix refers to filler typology. MH and AH are used for magnesium hydroxide and aluminum hydroxide, respectively. The second suffix is applied to distinguish the filler particles’ size. In particular, L and S are used for large- and small-sized fillers. As a reference, the batch MH-S refers to a sample modified with the magnesium hydroxide filler with small-sized particles. The TS code is used to identify the unfilled reference sample. A standard non-flame coating (STD) was also used to better appreciate the thermal insulation conducted by flame-retardant paints. Codes and FR compound specifications of all composite coatings investigated in the paper are summarized in Table 1.

### 2.3. Coating Characterization

The coded samples were analyzed by means of scanning electron microscopy (SEM, Quanta 450, FEI, Hillsboro, OR, USA) and X-ray diffraction (XRD, Bruker D8 Advance, Bruker, Billerica, MA, USA) to determine their morphology and crystal structure. The SEM analyses were performed in a low vacuum with an accelerating voltage of 20 kV. The XRD apparatus has a Bragg–Brentano θ–2θ configuration with CuKα radiation (40 V, 40 mA). XRD patterns were collected in the range 10° to 80° with a step of 0.1°/s.

In order to determine thermal and oxidative stabilities of FR coatings, thermo-gravimetric analysis (TGA) was conducted. Measurements were performed in air (100 mL/min) in the temperature range 30–1000 °C (scan rate: 10 °C/min), using a TA Instruments SDTQ 600 (New Castle, DE, USA) (balance sensitivity: 0.1 µg). Furthermore, in order to better assess if the loading of Mg(OH)_2_ and Al(OH)_3_ influences the adhesion of the coating, pull-off adhesion tests, according to ASTM D4541, were performed for all the investigated batches. A portable pull-off adhesion tester (Positest AT-M by Defelsko, Ogdensburg, NY, USA) was used to quantify the perpendicular force required to pull out a loading fixture away from its substrate (surface area glued with the substrate 78.5 mm^2^).

### 2.4. Flame-Retardant Property Measurement

The flammability test, adapted from EN ISO 119225-2, was performed on all coated panels. The procedure involved the exposure of the samples (placed in a vertical position) to a direct flame (of 20 mm, incident on the coated surface with an incidence angle of 45°). During the test, ignition time, flame spread and weight loss were recorded. Furthermore, a contact thermocouple was placed on the back of the specimen in order to acquire the temperature evolution on the opposite side of the fire exposure.

**Figure 2:**
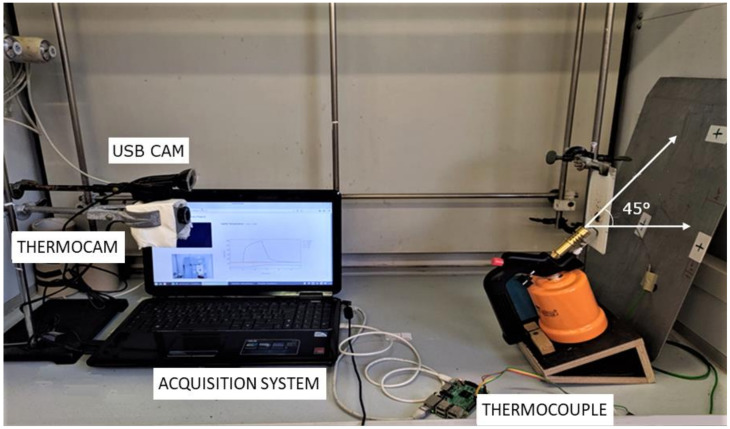
Burning system set-up.

The burning system set-up (Figure 2), according to the assembly proposed by [25], comprises the painted sample (75 mm × 140 mm) vertically fixed in a test hood chamber.

The center of the surface was exposed to a gas flame, supplied by a Bunsen burner, for 15 s and 30 s. The exit hole of the burner was placed at a distance of 20 mm from the testing surface with an incidence angle of 45°. Two replicas for each formulation were carried out. The coating flammability results were assessed, comparing burning time, temperature evolution and visual analysis of the surface.

## 3. Results and Discussion

### 3.1. Structural and Morphological Analysis

**Figure 3:**
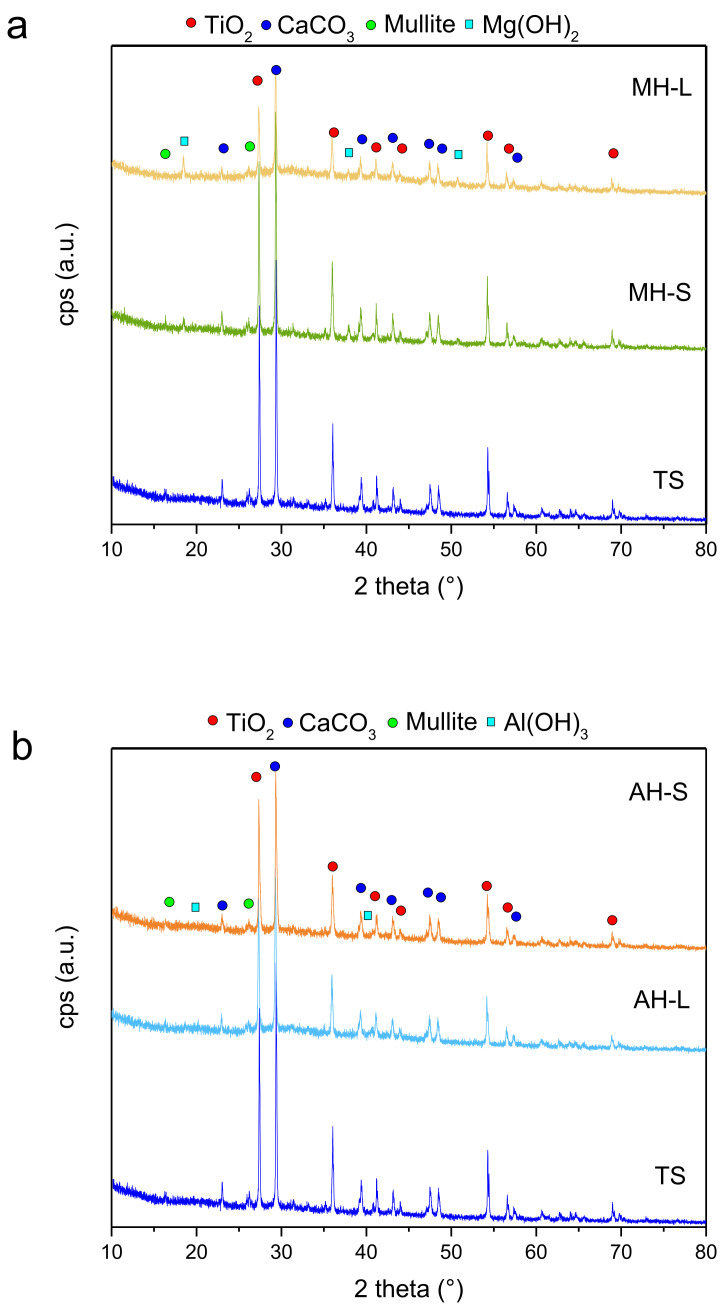
XRD of (**a**) MH- and (**b**) AH-filled coatings in comparison with the TS reference sample.

To investigate the obtained coatings’ structure, XRD analysis was carried out on all the studied samples, and is the results are shown in Figure 3. The filled coatings were compared with the TS reference sample.

All diffractograms show a clearly crystalline structure, mainly composed of two phases, TiO_2_ (JCPDS #89-552) and CaCO_3_ (JCPDS #1-83-1762). The less intense peaks of mullite (JCPDS #4-9-3667) are also noted. Mullite is an excellent reinforcer because of its light weight, high temperature resistance and noninflammability [26]. Regarding the filled products, it is possible to clearly distinguish the peaks of brucite Mg(OH)_2_ (JCPDS #7-0239) and bayerite Al(OH)_3_ (JCPDS #20-0011) in the MH (Figure 3a) and AH (Figure 3b) coatings, respectively.

Even if the intensity of the peaks of the MH-L sample is lower, indicating lower crystallinity of the coating, the peaks related to the added brucite are more intense and more easily visible than the MH-S sample. This indication is closely linked to the morphology of the additive, previously shown in Figure 3. In fact, the more intense and narrower peaks of the MH-L sample identify larger Mg(OH)_2_ crystallites than in the MH-S sample, represented by lower and wider brucite peaks. In AH coatings, such a noticeable difference is not present; the bayerite peaks are not clearly visible and widened, confirming that the FR additive crystallites have a similar size, although the samples have a different degree of packing, as reported by the SEM analysis of the fillers in Figure 1.

**Figure 4:**
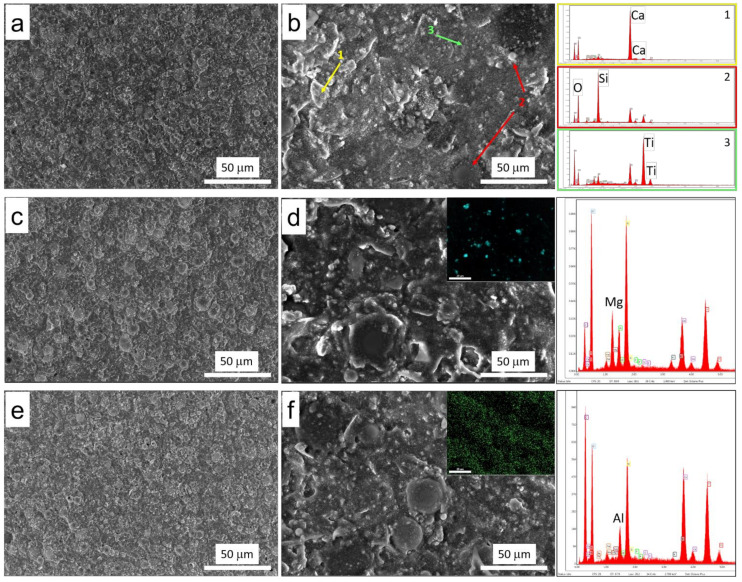
SEM and EDX of (**a**,**b**) TS, (**c**,**d**) MH-S and (**e**,**f**) AH-S coatings.

In order to assess the dispersion of the fire-resistant constituents, all the composite coatings were examined by coupling SEM and EDX analysis. Figure 4 shows the results for composite coatings modified with magnesium and aluminum hydroxide (MH-S and AH-S, respectively). As a reference, the as-received unmodified coating, TS, is also reported.

At low magnification (Figure 4a), the TS sample is homogeneous, with no evidence of microdefects or fractures. The different components are distinguishable, but uniformly distributed in the polymer matrix. Different morphologies composing the texture are evident. At higher magnification (Figure 4b), evaluating the peaks observed in EDX analysis, the main constituents, previously detected by X-rays (Figure 3), can be identified. In particular, the agglomerates of irregular shape, with a size between 10 and 30 µm, indicated by the yellow arrow and by the number 1, identify CaCO_3_. The spherical particles of two different grain sizes, ~25 µm and ~10 µm, indicated by the number 2 and the red arrows, are the hollow microspheres, while the nanometric particles uniformly incorporated in the matrix and marked with the green arrow and the number 3 are TiO_2_. The other peaks detected by the EDX are C, deriving from the organic matrix; Na, P and K, from minor organic and inorganic additives; and Al, from the microscope stub.

Observing the SEM images of the filled coatings, there are no substantial differences from the reference sample. In the EDX analysis, the peaks of Mg and Al elements for MH-S (Figure 4c,d) and AH-S (Figure 4e,f) batches, respectively, are clearly evident. This result highlights that the flame retardant has suitable compatibility with the acrylic matrix. The coating morphology of MH-S and AH-S is homogenous, without microdefects or cracks. The fillers are well interconnected with each other and with the polymer matrix that acts suitably as a binder. Furthermore, the reported mapping of Mg (cyan marker in corner of Figure 4d) and Al (green marker in corner of Figure 4f) elements for MH-S and AH-S, respectively, confirms that the fire-retardant fillers have favorable dispersion in the polymer matrix, indicating their suitability for this formulation. The high concentration of Al in the AH-S sample, observed by EDX analysis, is justified considering that Al is present not only as Al(OH)_3_ filler, but also as mullite silicoaluminate, as evidenced by the XRD analysis previously described. For this reason, the Al peak is also visible in the characteristic EDX of MH-S and TS samples. Similar considerations were observed for all other batches (not included here for the sake of clarity).

Furthermore, in order to better assess if the loading of Mg(OH)_2_ and Al(OH)_3_ influences the adhesion of the coating, Table 2 shows the pull-off strength for all the investigated batches. It is noted that the TS batch shows a pull-off strength of 4.82 MPa. The addition of the FR compounds leads to a slight reduction in strength, roughly in the range of 10% to 20% (the effect is more marked for AH-S and AH-L batches). In any case, this difference can be considered not significant, because the average data fall within the experimental dispersions.

### 3.2. Thermal Oxidation Decomposition Behavior

**Figure 5:**
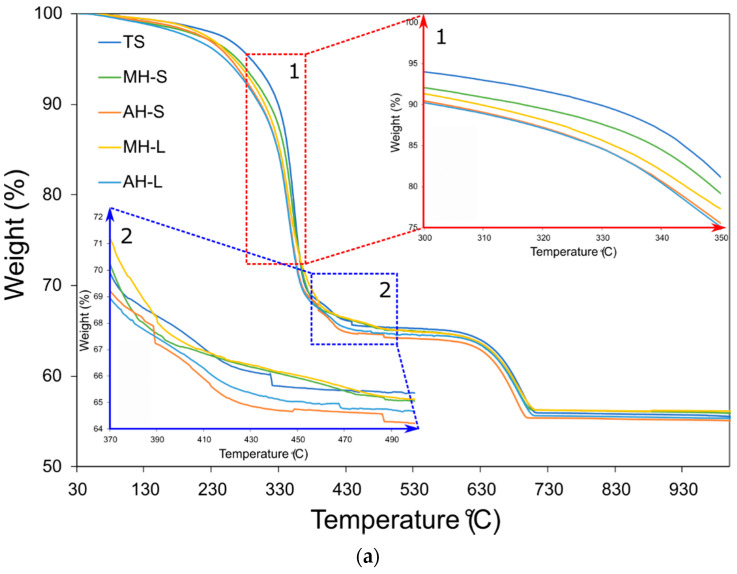

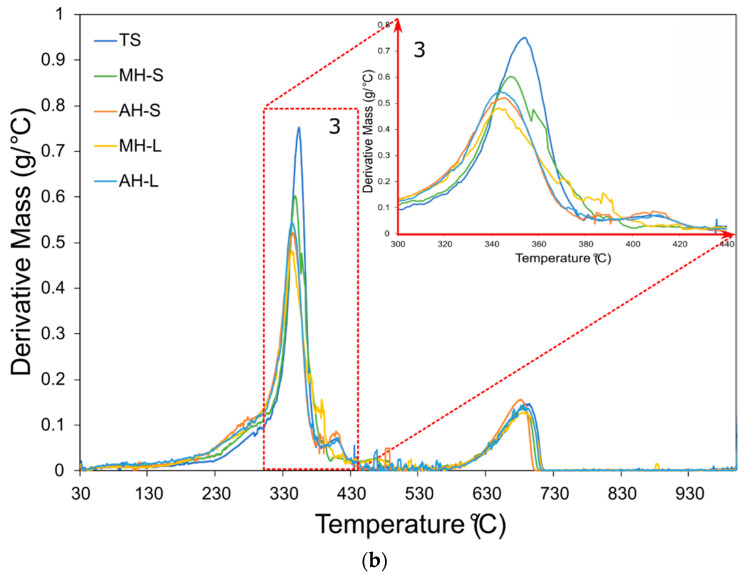
Thermograms of the composite coatings’ (**a**) weight loss (%) and (**b**) derivative mass (g/°C) at increasing temperatures.

At first, based on its applicability to fire safety in the shipbuilding sector, in order to evaluate the oxidative thermal stability of the coatings, thermogravimetric analysis (TGA) was carried out under air flow (100 mL/min) at a heating rate of 10 °C/min. Figure 5a,b compare the weight loss and the related derivative mass of all batches, respectively. All batches show similar weight loss trends. This is attributable to the similar constituent ratios in order to preserve the ratio between titanium oxide, hollow microspheres, carbonates, polymeric binders and active FR compounds.

Evaluating the trend of the curves in Figure 5a, two thermal degradative steps can be identified at about 350 °C and 680 °C.

The first most relevant weight loss, occurring at a lower temperature, can be identified as the main degradation step (it occurs in the range 300–430 °C), leading to the formation of a primary carbonaceous char [27]. The slight weight loss at 500 °C can be ascribed to a further char oxidation decomposition of the acrylic matrix and the organic compounds [28]. Afterwards, under oxygen atmosphere, the formation of ash and carbonized material as residual products takes place. The image in the left corner of Figure 5a (frame 1 in the figure) shows a magnification of the curves in the temperature range 300–350 °C, corresponding to the onset of the degradation phenomena listed above. It can be noted that the unmodified reference TS batch has the best thermal stability, triggering the weight loss in a higher temperature range than the other batches.

Analyzing all the modified samples, a progressive reduction in the activation temperature of the degradative phenomenon occurs. The samples modified with aluminum hydroxide (AH-L and AH-S) show premature weight decay compared to the others. Similarly, the MH-S batch, modified with C1-MH commercial magnesium hydroxide, has thermal stability in this temperature range close to that of the base material (TS).

The dehydration process of the aluminum and magnesium hydroxides influences the weight decay in this temperature range. The reaction occurs at temperatures in the range of 180–230 °C for Al(OH)_3_ [12,29]. The decomposition reaction of Mg(OH)_2_ takes place in the range 200–400 °C [30]. Therefore, this favors a slight contribution to the weight reduction for coatings characterized by the presence of these additives.

These considerations are supported by the analysis of the peak in the derivative mass curve (Figure 5b and its magnification in frame 3). The magnitude of the peak allows us to identify the maximum rate of weight loss and the temperature at which this occurs. All curves, although with more or less marked differences, display a similar trend, thus indicating a compatible degradation mechanism for all of them. However, the degradation activation at higher temperatures for the TS and MH-S batches can be attributed to a better interfacial interaction between the coating constituents, which increases their thermal stability. Conversely, the other batches are characterized by a maximum of the thermal degradation phenomena (Figure 5b magnification in frame 3) in the range 340–345 °C, about 10 °C lower than the reference TS batch.

Furthermore, it can be noted that the larger-sized FR additives imply a premature thermal degradation compared to the smaller ones, in the order AH-L < MH-L < AH-S < MH-S < TS.

This behavior suggests low interfacial interaction between the filler and the matrix, which invalidates the attended thermal stabilization action offered by the active filler. Further considerations can be drawn by evaluating the evolution of the weight loss curves in the temperature range 370–500 °C (Figure 5a, frame 2). The plot in the corner can be identified as the tail of the occurred degradation of matrix and organic compounds, previously discussed. In this range of temperature, the residual oxidation decomposition of the polymeric char takes place. Secondary degradation reactions force the polymer cracking, achieving a plateau in the weight loss trend at about 65%.

Afterwards, in the temperature range 630–700 °C, a second abrupt weight decay of about 8–10% is observed. This behavior can be associated with the decomposition process of calcium carbonate into calcium oxide (CaO, as residue) and carbon dioxide (gaseous CO_2_) [31,32]. The residual of all samples is almost similar, equal to about 55%. This value is compatible with the acrylic matrix and organic additive content in the coating formulations.

In summary, Table 3 shows degradation at low and high temperatures (T1 and T2, respectively) for all batches. Al(OH)_3_– and Mg(OH)_2_-filled batches exhibit low T1 values compared to the unmodified one. The lowest values are observed for AH-L and MH-L at 343.65 °C and 343.96 °C, respectively, indicating an earlier thermal degradation of large particle-based coatings. The presence of large-sized filler particles likely involves a desegregation of the constituents with a detrimental effect on the thermal stability of the material. This deviation is less evident for T2. In this case, all filled batches evidence a similar temperature, about 687 °C, lower than that of the unfilled coating (except AH-S, which records the lowest temperature, 681.73 °C). Taking into account that the thermal transition at about 680 °C can be associated with the CaCO_3_ calcination, this result suggests that the nanoparticle size of the Al(OH)_3_ filler and, accordingly, of the produced Al_2_O_3_, strongly influences the oxidation temperature of calcite. Przekop et al. [32] observed a shift to lower calcite carbonization temperatures due to a particle size reduction of Al_2_O_3_–CaO–CaCO_3_ systems, increasing the Ca/Al ratio.

### 3.3. Flame-Retardant Performances

**Figure 6:**
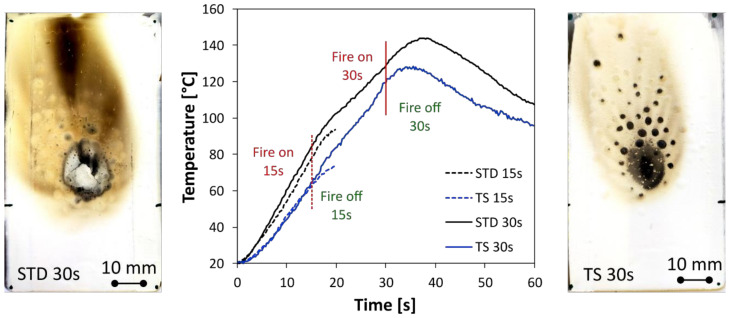
Fire test images and thermocouple temperature of STD and TS samples.

Preliminarily, a standard coating (STD), not flame-retardant, and a thermal insulating coating used as a reference (TS) were investigated. Figure 6 shows the thermocouple temperatures of STD and TS samples in fire tests after 15 s and 30 s. Furthermore, optical surface images for samples exposed to a direct flame for 30 s are also added for both coating batches.

The STD sample exhibits a relevant degradation at increasing flame exposition time. At 30 s, the coated surface is largely degraded. A wide carbonization along the flame direction is observed. At the same time, the temperature measured by the thermocouple, positioned behind the stainless steel plate, is 129.5 °C at 30 s of fire exposition, with a maximum of 143.7 °C observed at 37 s. This confirms that the protective action offered by this class of coating is not effective. The coating does not guarantee a barrier protection against the thermal flux induced by the flame source applied on the opposite surface. Given this concern, the addition of thermal active fillers serves to increase the flame stability and the protection of the coating in severe conditions of direct fire contact.

The TS specimen exhibits better flame stability than the STD one. By analyzing the evolution of the temperature, we can see that the former shows a better heat barrier action, as identifiable by the reached temperature, lower than the STD one. Furthermore, the coating surface for the TS sample exposed to a direct flame for 30 s shows that the suffering area is mainly localized in the zone where direct contact with the flame occurs. Some scattered and randomly dispersed combusted zones (black circles in the plot) in proximity of the flamed area can be also identified. These areas can be related to the charring of particular regions with a high concentration of acrylic resin [33].

**Figure 7:**
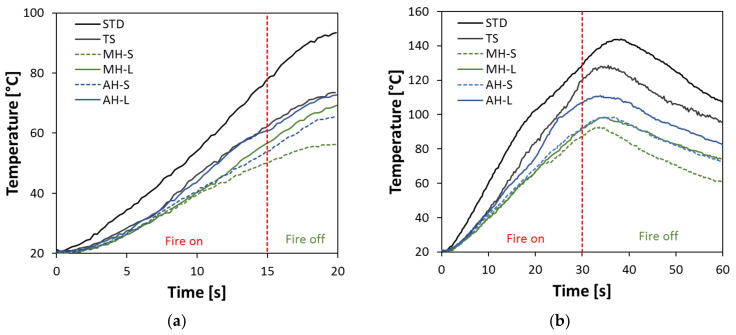
Back temperature evolution of stainless steel substrate for (**a**) 15 s and (**b**) 30 s of direct exposition to a flame for all coatings during fire tests.

In order to evaluate how the addition of the FR additives influences the flame-retardant capacity of the barrier TS coating, the evolutions of the temperature on the stainless steel support for fire resistance tests performed at 15 s and 30 s are summarized in Figure 7a and Figure 7b, respectively. In all figures, the fire on and fire off steps during the recording stage are highlighted. The vertical dotted red lines identify the moment the fire source is switched off, being 15 s and 30 s for Figure 7a and Figure 7b, respectively.

Preliminarily, evaluating the results reported in Figure 7a, it can be seen that the STD coating does not show a significant barrier protection. The chosen standard coating does not significantly hinder the thermal flow from the flame source to the metal support. This can be seen from the high temperature reached even at a short exposure time to the flame.

Conversely, all other batches, designed to offer a barrier action and flame resistance, show a much more gradual temperature trend with increasing time. All the specimens exhibit a similar trend, with a maximum of about 56–73 °C at about 20 s. In particular, it can be noted that the specimens with fine-grained FR additives (MH-S and AH-S) show better efficiency in terms of fire protection than the other specimens. Their flame-retardant action is activated after about 7–8 s, as evidenced by the curves of the MH-S and AH-S specimens, which begin to deviate from the other ones. When the fire is switched off (15 s), the temperature continues to increase due to thermal inertia, without showing a stabilization condition at the end of the measurement (20 s). This suggests that longer exposure times are required to allow the FR additives to fully perform their protective action. The other FR-functionalized batches do not exhibit significantly different behavior than the TS one, indicating that coarse-grained fillers may require longer activation times to supply effective fire protection.

Further considerations can be drawn by analyzing Figure 7b, depicting the results of the 30 s fire test. The results confirm the best fire resistance for composite coatings functionalized with small-sized FR additives. Furthermore, the most critical test conditions (30 s of fire exposition) led to the achievement of a temperature for the stainless steel support significantly higher than that achieved for 15 s of fire exposition. All tests exhibited a maximum temperature at about 35–40 s. Furthermore, thanks to the addition of FR, the maximum temperature compared to the TS sample was reduced by 17–30 °C for large-sized FR filler and by approximately 30–35 °C for small-sized FR filler.

Al(OH)_3_ and Mg(OH)_2_ hydroxides contribute to dissipate the thermal energy supplied during firing. According to Equations (1) and (2), the hydroxides decompose with an endothermic dehydration reaction, releasing water vapor [12,13]. The main difference is related to the temperature required to activate the reaction and the related endothermic reaction enthalpy. In particular, Mg(OH)_2_ dissociates at a higher temperature (332 °C) than Al(OH)_3_ (180 °C), involving absorption heat about 32% higher (1389 J/g [15] and 1050 J/g [16] for magnesium and aluminum hydroxides, respectively). During this process, the filler particle size plays a key role. A small and well-distributed FR additive favors thermal exchange, thus exalting the fire resistance capacity of the coatings.

**Figure 8:**
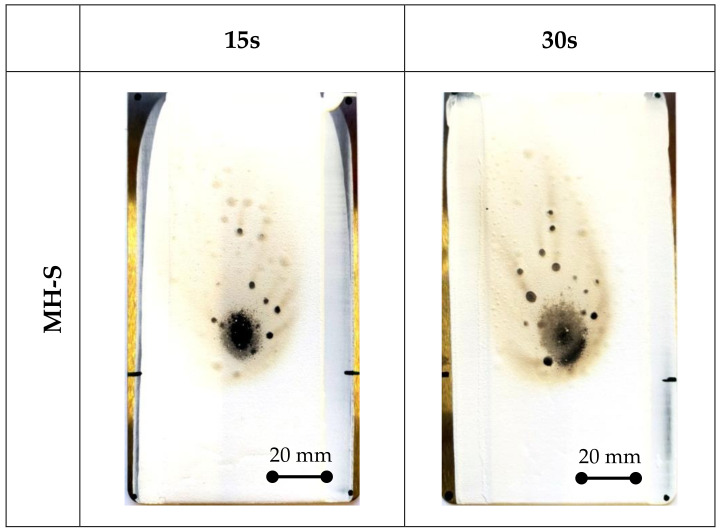

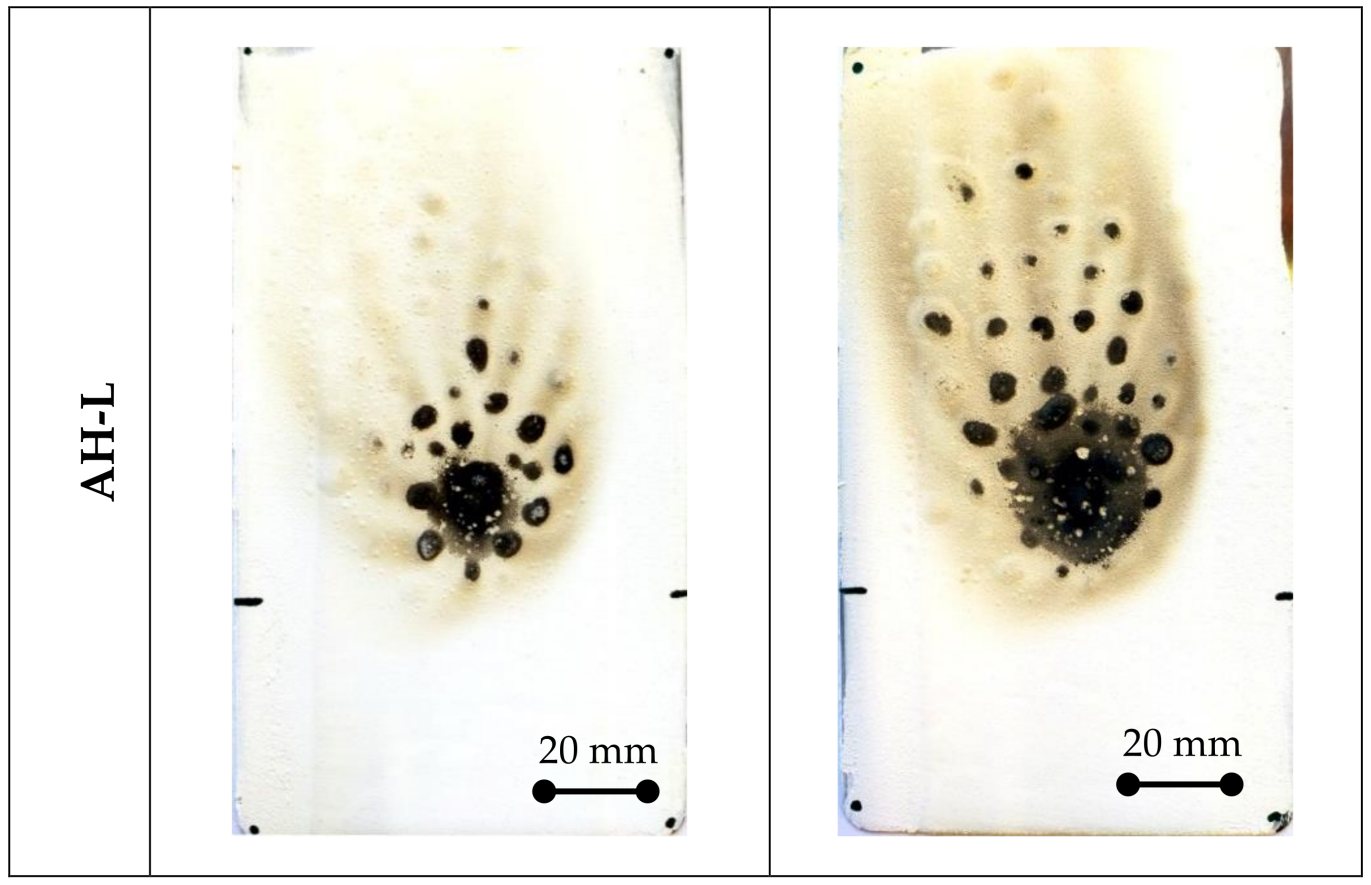
Fire test images of flame-retardant samples MH-S and AH-L.

The visual results of the fire resistance tests of MH-S and AH-L batches at 15 s and 30 s are compared in Figure 8. The coating surfaces for the composite coatings with a combustion time of 15 s (left column in Figure 8) highlight some visual modifications induced by the addition of the FR additives. Already at a short exposure time, all samples show a slight surface degradation due to the exposition to the direct flame. An ellipsoidal browning of the surface coating can be identified. Furthermore, some black dimples are randomly located in the degraded area due to the localized charring of some regions with higher acrylic resin concentrations.

The TS sample, without the FR additives, has an acceptable level of fire resistance. The specimen has a fire damage area extended on the upper side of flame source. The coating area in direct contact with the flame is black and circularly shaped. Some superficially combusted lines (dark brown rivers) extend radially from this central zone (not included here for the sake of clarity).

The addition of the metal hydroxide affects the flame-retardant properties of the coating. It is worth noting that, depending on the FR size, a beneficial or detrimental action to the fire resistance can be identified. Considering an FR sample with a small particle size (MH-S) exposed for 15 s, the surface modification of the coating induced by the flame exposition for 15 s is quite limited. Instead, when using a large-sized FR (AH-L sample), there are no obvious advantages. The damaged area is almost similar to the unmodified TS sample.

Further information can be gained by analyzing the coating surfaces exposed for 30 s to the fire (right column in Figure 8).

Evaluating the TS sample (Figure 6), a longer exposure time to the fire imposes an evident extension to the damage area. The damage area extends along almost the entire upper portion of the sample, indicating that the degradation phenomenon has superficially enlarged, due to both the flame spread and the higher temperature reached on the surface. Evaluating the modified specimens (MH and AH batches), it is found that only the specimen with the small-sized magnesium hydroxide addition (MH-S) shows excellent fire stability. The damage distribution is restrained and relatively homogeneous. Conversely, all the aluminum hydroxide specimens show a sudden decay in performance due to a longer exposure time to fire (see AH-L, right column image in Figure 8), exhibiting fire resistance after 30 s of exposure, quite similar to the unmodified TS sample (Figure 6).

This behavior can be justified considering that aluminum hydroxide adsorbs less energy during the decomposition process (1050 J/g [15]) in comparison with magnesium hydroxide (1389 J/g [16]). The endothermic decomposition withdraws heat from the substrate, slowing the rate of thermal degradation of the coating. However, according to Beyer et al. [15], the Al(OH)_3_ metal hydroxide influences the degradation at a lower temperature than Mg(OH)_2_, triggering the structure protection process earlier than magnesium hydroxide. The activation of the dehydration process of Mg(OH)_2_ and its protective action occur at higher temperatures. This implies that its contribution to the coating’s fire resistance becomes more relevant as the temperature approaches the dissociation temperature of the hydroxide. The MH-S and MH-L batches avoid flame spreading better than the TS one. It is also worth considering that in both of the fillers’ reactions, the decomposition products are non-toxic and the mineral phases, especially MgO, are alkaline, reducing the probability that acid and corrosive gases are released by combustion.

Even if a low filler percentage is used, in this exposition range, the incorporation of such hydroxides has a beneficial effect on the fire protection of the coating. Based on the promising results, future developments will focus on the combined use of the studied fillers in order to take advantage of the higher amount of heat absorbed, exploit the different activation temperatures of the two hydroxides and cover a wider range of intervention temperatures.

## 4. Conclusions

In this work, we investigated the use of low percentages (2wt%) of magnesium (MH) and aluminum (AH) hydroxides as flame-retardant fillers to evaluate their degree of protection on a stainless steel substrate. Two different particle sizes were chosen for each filler, large and small. The implemented coatings were compared with a standard non-flame-retardant coating via flame tests and by visually assessing the consequences of combustion at 15 s and 30 s. In addition, the insulation capacity was analyzed by measuring the temperature evolution behind the metal support.

- The coatings have been thermally characterized and the addition of the fillers decreases the activation temperature of the combustion process (~345 °C vs. ~354 °C for the filled specimens and the unfilled one, respectively).
- The presence of larger-sized particles disadvantages the flame resistance, highlighting a wider area affected by the combustion. In such a context, the AH-L sample has a similar behavior to the unfilled TS one. The degree of thermal insulation is higher for samples with small-sized fillers, particularly at 30 s of fire exposition.
- The order of flame protection can be identified as: MH-S > MH-L > AH-S > AH-L > TS. In particular, MH-S exhibited a back temperature of ~85 °C vs. ~120 °C for the TS sample and 130 °C for the non-flame-retardant coating (STD).

What emerged from the study, comparing the two types of fillers, is that the AH batches show a lower intervention temperature but a lower level of protection, involving, in the endothermic dehydration reaction, a minor amount of heat compared to the MH batches. However, in both cases, the small percentage of used filler is able to protect the structure at a short time of fire exposure. Future developments will require the use of both of these non-toxic and non-corrosive fillers to broaden the temperature range of action and optimize flame resistance.

## Figures and Tables

**Table 1:** Codes and FR compound specifications of composite coatings.

Code	FR Compound		TR Specifications
	Type	Content (wt%)	
STD	//	//	//
TS	//	//	//
MH-S	Mg(OH)_2_	2	Acros Organics—USA
MH-L	Mg(OH)_2_	2	Kisuma Chemicals BV—The Netherlands
AH-S	Al(OH)_3_	2	Sigma-Aldrich—USA
AH-L	Al(OH)_3_	2	Synthesized by precipitation

**Table 2:** Pull-off strength.

Code	TS	MH-S	MH-L	AH-S	AH-L
Pull-off strength (MPa)	4.82 ± 0.65	4.43 ± 0.56	4.66 ± 0.29	3.89 ± 0.28	3.97 ± 0.54

**Table 3:** Thermo-gravimetric parameters.

Code	T1 (°C)	Weight Loss at 530 °C (%)	T2 (°C)	Weight Loss at 750 °C (%)
TS	354.79	34.71	691.80	44.07
MH-S	348.63	35.00	686.78	43.77
MH-L	343.96	35.08	687.89	43.75
AH-S	345.74	35.84	681.73	44.62
AH-L	343.65	35.40	686.13	44.38

## Data Availability

Data are contained within this article.
